# Significant Features for Human Activity Recognition Using Tri-Axial Accelerometers

**DOI:** 10.3390/s22197482

**Published:** 2022-10-02

**Authors:** Mohamed Bennasar, Blaine A. Price, Daniel Gooch, Arosha K. Bandara, Bashar Nuseibeh

**Affiliations:** 1 School of Computing and Communications, The Open University, Walton Hall, Milton Keynes MK7 6AA, UK or; 2 Lero, Irish Software Research Centre, Tierney Building, University of Limerick, V94 NYD3 Limerick, Ireland

**Keywords:** tri-axial accelerometer, human activity recognition, feature selection, activity of daily living, Physical Activity, classification

## Abstract

Activity recognition using wearable sensors has become essential for a variety of applications. Tri-axial accelerometers are the most widely used sensor for activity recognition. Although various features have been used to capture patterns and classify the accelerometer signals to recognise activities, there is no consensus on the best features to choose. Reducing the number of features can reduce the computational cost and complexity and enhance the performance of the classifiers. This paper identifies the signal features that have significant discriminative power between different human activities. It also investigates the effect of sensor placement location, the sampling frequency, and activity complexity on the selected features. A comprehensive list of 193 signal features has been extracted from accelerometer signals of four publicly available datasets, including features that have never been used before for activity recognition. Feature significance was measured using the Joint Mutual Information Maximisation (JMIM) method. Common significant features among all the datasets were identified. The results show that the sensor placement location does not significantly affect recognition performance, nor does it affect the significant sub-set of features. The results also showed that with high sampling frequency, features related to signal repeatability and regularity show high discriminative power.

## 1. Introduction

Increasing Physical Activity (PA) is a significant part of daily life, which can have beneficial health effects [1]. Physical inactivity has been identified as one of the top four risk factors for global mortality: inactive people are vulnerable to several health conditions such as high blood pressure, diabetes, cardiovascular diseases and cancer [2]. Physical inactivity is a significant concern amongst the globally ageing population. In 2017, there were twice as many children under the age of 15 as the number of people aged 60+ in the world, but by 2050, these numbers are expected to be equal [3].

Managing the expected demand in social and health services will need to harness opportunities from new technologies. Ambient Assistive Living (AAL) technology has the potential to relieve some pressure from both families and social services, and provide more care in the home and community, which will reduce national health and care spending and has the potential to improve wellbeing [4]. Monitoring the activities of older people during their daily life can improve their safety, autonomy and quality of life [5]. 

In the past, a common way of assessing PA was the use of questionnaires, such as the Physical Activity Scale for the Elderly [6]. Such questionnaires are limited as they cannot provide accurate continuous data of lived experience, relying instead on individuals’ rationalization of their behavior.

This has led to a great deal of interest in the potential of wearable technologies for monitoring PA. Currently, the accelerometer and gyroscope are the most widely used wearable sensors for PA monitoring. They have been used in a variety of applications, including heath monitoring, fall detection [7], human movement monitoring [8], gait analysis [9], disease progression assessment [10], and activity recognition [11]. Accelerometers are a popular choice of sensor due to their practicality, low cost and reliability, and have been employed in many systems to classify Activities of Daily Living (ADL), also known as Human Activity (HA) [5]. Wearable technology including smart phones is an ideal platform for monitoring ADL, because it can be deployed outside the controlled environment in free-living conditions, it has a relatively low cost and high acceptability/compliance. However, computer algorithms are still needed to translate the acceleration signals into different activities, this process is called activity recognition. 

Activity recognition is also employed in industrial applications. According to the Industry 4.0, monitoring and recognising human activities in manufacturing space is the most important component to integrated into a cyber-physical system [12]. In manufacturing space that is chaotic and full of moving objects, employing computer vision techniques to recognise human activities is very challenging, especially for craft industrial scenarios. Accelerometers are one of preferred choices for monitoring human activities within industrial areas [13].

According to the literature, accelerometers have been placed on many positions on the body, such as the waist (belt), wrist, upper arm, chest, pelvis, thigh, and ankle [14]. Several studies have been conducted to assess the effect of sensor placement on recognition accuracy [15,16,17]. The wrist has shown the best performance for recognition compared with other positions [14]. Furthermore, it has been reported that placing an accelerometer on the wrist is the optimal placement position because it is most preferred by participants [14]. 

Most activities in real life situations consist of several complex motions, which means that simple wearable sensors may not be enough to recognise these activities. However, they are very effective for recognising simple activities [18]. 

In this paper, a comprehensive list of features is extracted from 3-axial signals, which includes a set of features that never been used before for human activity recognition. Filter feature selection method is used to investigate the significance of each feature for the activity recognition. The comparison involves comparing the effects of sampling rate, sensor placement, and complexity of activities on the significance of the features, and on the classification performance. 

### 1.1. ADL Recognition with Accelerometers: Background and Literature Review

Typical ADL recognition using an accelerometer is usually achieved by a task that consists of several steps: signal pre-processing, feature extraction, dimensionality reduction and classification [19]. The classification is not performed on every single data point in the acceleration data; instead, the signal is segmented using a sliding window with some degree of overlap. This minimises the effect of noise and reduces the computational cost of the learning algorithm. There is no agreement about the window size, as each activity has a different duration. Therefore, there should be a trade-off: a small window is not enough for a long activity, while a large window may contain more than one activity. A variety of window lengths have been used in the literature [20]. The feature extraction step involves calculating attributes that are able to capture the patterns over a sliding window. The features can be categorised into three: time domain, frequency domain, and time-frequency domain. Various features have been used for the ADL recognition task in the literature, starting from simple statistical methods (e.g., signal mean, signal standard deviation, correlations, etc.) to frequency domain features. However, there is no consensus on the best features that should be extracted and used for classification [21]. Using a large number of features does not necessarily improve the classification. Moreover, using high dimensional data will increase the computational burden and make the classifier model complex. Therefore, dimensionality reduction techniques are usually used to reduce the number of features. Feature selection is a dimensionality reduction used to identify the informative features which are relevant to the classification task and eliminate redundant and irrelevant features.

It is well known that classification accuracy can be enhanced through reducing dimensionality [22]. In ADL recognition, using relevant high-quality features and excluding irrelevant and redundant features is essential for improving classification accuracy and reducing the computational cost. Therefore, in order to achieve the best performance, the dimensionality (number of features) should be reduced as much as possible. 

The two main techniques used for dimensionality reduction are feature extraction and feature selection. Feature extraction involves the creation of a new feature space as a combination of the original features. The most common methods are Principal Component Analysis (PCA) [23], Linear Discriminant Analysis (LDA) [24] and Singular Value Decomposition (SVD) [25]. Feature selection, on the other hand, involves the selection of the best subset of the original features. It can be categorised in terms of its relation to the classification algorithm, such as filter, wrapping and embedded methods. Wrapping methods are classifier dependent and use classification accuracy as a measure of the performance of the selected subset of features. These methods are very specific to the classification algorithm used for the evaluation. Embedded methods are also classifier dependent methods; they are part of the classifier. Unlike the previous methods, filter methods are classifier independent. They measure the quality of selected features without the involvement of a classifier algorithm [26].

Researchers usually collect their own data to train and validate their proposed system. This makes it very difficult to compare the performance of the different proposed techniques in an objective way [27].

Accelerometers have been used in many ADL recognition approaches in the literature. However, each study focuses on different activities and extracts diverse features, which makes it difficult to compare performance between studies. Many machine learning techniques have been used for ADL recognition, such as Random Forest [28], Support Vector Machine (SVM) [29], Decision Trees [30], Hidden Markov Models [31], and Bayesian Networks [29]. Data can be collected at different sampling frequencies; however, a 20 Hz sampling frequency is enough for capturing simple activities such as walking, standing, and sitting which are fairly regular [32]. However, a low sampling frequency may hide significant details about complex activities that have less regularity in the acceleration signals [33]. 

Feature selection has also been employed for activity recognition applications. Preece et al. (2009) [34] compared the classification accuracy of fourteen feature extraction methods used in the literature. A specific dataset was collected for this study using three EBT tri-axial accelerometers attached to the waist, the thigh and the ankle. The results showed that Fast Fourier Transform (FFT) features perform the best. The authors studied the feature sets individually. 

Relief-F and Sequential Forward Floating Search (SFFS) wrapping methods were applied to a range of features from the literature [35]. The authors collected the data using one Freescale MMA 7260 tri-axial accelerometer attached to the waist using a custom-made belt. Statistical features (mean, variance, maximum acceleration difference) and spectral features were used. They employed two classifiers to evaluate the selected subset (K-Nearest Neighbor (KNN) and Naïve Bayes). They identified a variance of the x,y,z axis, entropy of x,y,z energy uncorrelated, window mean x,y,z, mean trend x,y,z and fluctuation analysis coefficient as the most significant subset. 

Capela et al. (2015) [19] used three filter feature selection methods: Relief-F, Correlation-based Feature Selection (CFS) and Fast Correlation Based Filter (FCBF). The selected feature sets were evaluated using several classification algorithms. In this research, gyroscope data was included in the analysis, and 76 features were extracted. The authors used SVM, J48 and Naïve Bayes classifiers to evaluate the performance of the selected subsets. The selected subsets were a collection of accelerometer and gyroscope features. 

Atallah et al. (2010) [36] used six tri-axial accelerometers to collect data. This number was chosen, as it was considered appropriate to study the ideal location for the sensor. They used a set of features consisting of statistical time domain features and features based on FFT. Relief-F, Simba and minimum Redundancy Maximum Relevance (mRMR) methods were employed to assess the significance of each feature. The performance of the three methods was very similar in terms of classification accuracy. They also agreed that the total FFT energy was the most relevant feature. 

Chernbumroong et al. (2011) [37] used an eZ430-chrons watch to collect data with a sampling rate of 33 Hz. The study focused on five activities: sitting, standing, lying, walking and running. The CFS method was used to select the features that highly correlated to the class label. The C4,5 classifier and Artificial Neural Network were used for the classification. The study did not specify which features were ranked as the most significant. 

Suto et al. (2017) [38] used nine feature selection methods to rank the extracted features. The authors focused on the most common features used by previous studies. Three classification algorithms (artificial neural network, KNN, decision tree) were used to evaluate the performance of the selected features, using the wearable action recognition database [39], which is collected from 20 participants who were asked to perform 13 common activities. The authors did not explain the evaluation criterion. The features have been investigated for each participant individually which may make the findings subject to sensor placement and orientation. The results were not consistent between the participants and the employed methods and classification algorithms.

Rosati et al. (2018) [40], divided the time domain and frequency domain features into two sets (FeatSet_A, and FeatSet_B), and evaluated the performance of each set on activity recognition. The study employed a data set that was collected using IMUs from 61 healthy participants as part of this study. A 5 s sliding window with overlap of 3 s was used to segment all accelerometer and gyroscope signals. A genetic algorithm was employed to perform feature selection and minimise each set. The SVM classifier was employed to classify the achieved 97.1% accuracy; the results also showed FeatSet_B out performed FeatSet_A, where FeatSet_B comprises a number of zero crossing, positive and negative peaks, and mean value, standard deviation, maximum, minimum, and range of duration for positive, negative, and total peaks.

Orlov et al. (2019) [41] employed the Relief-F algorithm to rank the features, where 54 features were extracted from the accelerometer signals. This study extracted only the simple statistical time domain features such as mean, standard deviation, and skewness. The authors reported improvement of about 1% on the classification accuracy after they applied the feature selection and reduced the number of features. They also report a 4-fold improvement in the processing time of the classifier.

Ahmed et al. (2020) [42] employed the SFFS method to study the significance of the features. The study focused on statistical and frequency features only. The authors employed one publicly available dataset to test their approach. Both accelerometer and gyroscope data were used to extract 138 features. The reported results showed that the optimal subset consists of 18 features which were extracted from the 3 axes. The common features between the axes are mean, standard deviation, skewness, and kurtosis. The rest of the features were different between the axes. The finding of this study is very specific to the SVM classifier and cannot be generalized.

Finally, Thakur et al. (2022) [43] employed Guided Regularized Random Forest (GRRF) feature selection method to analyse the significance of the smart phone sensor feature for HA recognition. 20 features were extracted from 3-axial signals. They include statistical, time domain, and frequency domain features. In this work, Random Forest (RF) as a feature selection algorithm. The results showed that reducing the set of features by removing the irrelevant and redundant features improved the classification accuracy and allow SVM classifier to outperform deep learning-based approach on the classification task. However, the authors did not list the most significant subset of features that was used to generate that accuracy. 

### 1.2. Contribution

Most of the previous studies extracted limited sets of features. Moreover, they used a limited set of features, and most of them employed just one specific dataset, which was collected as part of the study. The missing of the collected datasets and other key details of those studies does not allow us to evaluate the proposed approach against some of those studies.

In contrast, this paper extracts a comprehensive set of features from accelerometer raw data, some of which has never been used for this application before. Then, a framework is employed to measure the significance of the features from four publicly available datasets. These datasets have different sampling frequencies and different sensor placement locations; we have used them to comprehensively investigate the significance of the features for the classification. The investigation was performed at the study level; the significance of the features which were extracted from all the participants’ data have been analysed together. This framework consists of several steps and employs a state-of-the-art feature selection method: The Joint Mutual Information Maximisation (JMIM) method [22]. The paper also investigates the influence of sampling frequency, sensor placement location, and complexity of activities on the significance of the features. Another distinction from previous work, where researchers collected their own data, is that publicly available datasets have been used in this study to compare the features objectively and make the results reproducible.

The rest of the paper is organised as follows. Section 2.1 describes the datasets, Section 2.2 explains the experimental setup, Section 3 presents the results, and Section 4 discusses the results and concludes the paper.

## 2. Materials and Methods

### 2.1. Datasets 

To define the significant features for the activity recognition task using accelerometers, an analysis was performed using four different datasets with various characteristics. Two datasets are publicly available from the UCI machine learning repository [44], and the other two from [45,46]. Table 1 lists the characteristics of each dataset. These datasets have been previously used in activity recognition research [46,47,48,49,50]. They were selected to be able to test the selected features with a variety of placement locations, sampling frequencies, the overall performance of the activity recognition task and the inclusion of simple and complex activities in the testing datasets. 

#### 2.1.1. Wireless Sensor Data Mining Dataset (WISDM)

This dataset was collected in a controlled laboratory environment using an accelerometer sensor on a smartphone placed at the waist. The data was collected from 29 participants at a sampling frequency of 20 Hz. The data consisted of six activities (walking, jogging, ascending stairs, descending stairs, sitting and standing). No demographics about the participants were provided. 

#### 2.1.2. Heterogeneity Activity Recognition Dataset (HARDS)

The HARDS dataset (also called HHAR) has been collected using a variety of smartphones and smart watches from nine participants (age range 25–30 years) in a controlled setting using specific routes outside the laboratory. All of the participants were asked to perform five minutes of each activity following a given script. Several sampling frequencies were used to collect the data based on the device used. In this paper, only the part of the data collected using Samsung and LG smart watches were used. The watches used sampling rates of 100 and 200 Hz, respectively, to collect the data. Six activities were monitored (walking, biking, sitting, ascending stairs, descending stairs and standing). 

#### 2.1.3. Smartphone-Based Recognition of Human Activities and Postural Transitions Dataset (SBRHA)

A group of thirty participants (age range 19–48 years) were recruited to collect this dataset in a controlled environment. They were asked to perform six activities (walking, laying, sitting, climbing up the stairs, climbing down the stairs and standing). A smartphone placed on the waist was used to capture the activities at a sampling frequency of 50 Hz using the built-in tri-axial accelerometers and 3-axial gyroscopes. In this study, we are focusing only on the data from accelerometers. 

#### 2.1.4. Physical Activity Monitoring for Aging People (PAMAP2)

This dataset contains 12 different activities. Nine participants (1/9 female, total age range of 24–32 years) were recruited to collect this data using three Inertial Measurement Units and heart rate monitoring in a controlled environment outside the laboratory. 

One inertial measurement unit (IMU) was placed on the wrist on the dominant arm, another on the chest, and the last one on the dominant side’s ankle. The IMU is a device that consists of triaxial accelerometers, triaxial gyroscope, and triaxial magnetometer. The gyroscope is known for it is tendency to drift over time even when the sensor is not rotating [51]. In this study, only data collected from the accelerometer was used in this study; The data was collected at a frequency of 100 Hz. This dataset has been chosen to study the effect of sensor placement location on the predictive power of the features. 

### 2.2. Evaluation Framework 

**Figure 1:**
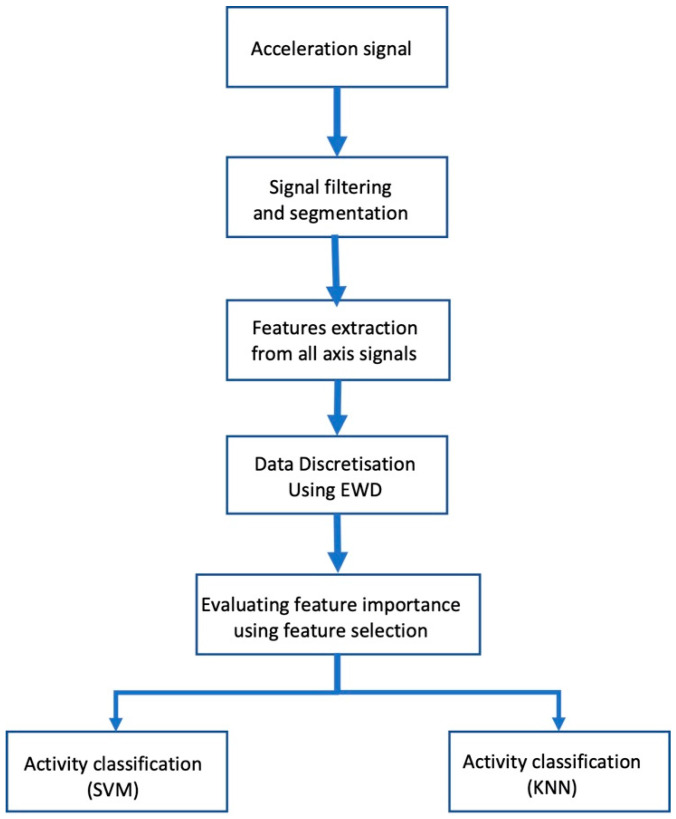
Feature significance evaluation framework.

The evaluation framework used in this study is shown in Figure 1; some of the steps are described in more detail below.

#### 2.2.1. Pre-Processing 

It has been reported in the literature that the best classification accuracy can be achieved using a sliding window between 2.5–3.5 s [52]. Therefore, each acceleration component (signal) is segmented using a 2.5 s sliding window. Each window is considered sequentially. Each observation (2.5 s) in the raw data is considered as an occurrence. For example, walking for five seconds is considered as two occurrences. An Infinite Impulse Response (IIR) high-pass filter was employed on each window to remove gravitational contributions.

#### 2.2.2. Feature Extraction 

Raw accelerometer data cannot be used directly by most ML algorithms. Features that measure the characteristics of the signal windows are extracted to capture the patterns among the data. Since this study investigates accelerometer signal features, the decision was made to extract most of the features that were reported in the literature. Time domain and frequency domain are commonly used measures as features for accelerometer data. In addition to the common features, features across the domain boundaries were included, which are not commonly used for activity recognition, such as:

- Recurrence Quantification Analysis (RQA) [53]: Recurrence is an essential property of any dynamical system. It is used to describe the behavior of the system in phase space. Recurrence Plots (RPs) are a method used for visualizing the recurrence behavior of dynamical systems [49]. Assume that a dynamical system is described in phase space with a set of trajectories $x_{i}^{\to },\;i=1,2,\ldots ,N$, these vectors are used to describe a quantity of parameters. The development of the system state is described by series of the vectors. The dynamical system can be represented by recurrence matrix R(i,j):
  $$R\left(i,j\right)=\Theta \left(\varepsilon -\|x^{\to }\left(i\right)-x^{\to }\left(j\right)\|\right)\;i,j=1,2,\ldots .,N$$
  where Θ: ℝ→(0,1),  N is the number of considered states, and ε are predefined distance. Ε is essential as the system usually recurs exactly visiting the same states.
  A periodic system is characterized by long and non-interrupted diagonals in RPs. The vertical distance between these diagonal lines reflects the period of oscillation. While in the chaotic system, diagonals are formed in RP, but shorter than periodic systems. Uncorrelated stochastic signals generate RP with many single points. Therefore, the shorter the diagonals in RP, the less predictable the system. Therefore, each activity is expected to have its diagonal characteristics.
  In order to quantify the characteristics of the recurrence plots and go beyond just visualizing them, RQA is used to produce quantitative measures based on the recurrence point density, diagonal lines, and vertical lines. Four variables for the quantification by RQA:
  1. Recurrence Rate (RR): The percentage of recurrence points in a recurrence plot. It is equivalent to the probability that a specific state will recur to its ε neighbourhood.
    $$\mathrm{RR}=\frac{1}{N^{2}}\overset{N}{\underset{i,j=1}{\sum }}R_{i,j}$$
  2. Determinism (DET): The percentage of recurrence points in a recurrence plot that form the diagonal lines of minimal length $l_{min}$. Processes with chaotic behavior cause short or no diagonals, while deterministic processes lead to longer diagonals. Therefore, to measure the chaotic behaviour, the ratio between the recurrence points along the diagonal structures and the total all recurrence points.
    $$\mathrm{DET}=\frac{\sum_{i=l_{min}}^{N}l\;P\left(L\right)}{\sum_{i=1}^{N}l\;P\left(L\right)}$$
    where P(L) is the histogram of diagonal line of length L.
  3. Entropy (ENTR): The Shannon information theory entropy of the probability distribution of the diagonal line length.
    $$\mathrm{ENTR}=-\overset{N}{\underset{l=l_{min}}{\sum }}p\left(l\right)\ln\left(p\left(l\right)\right)$$
    where $P\left(l\right)=\frac{P\left(l\right)}{\sum_{l=l_{min}}^{N}p\left(l\right)}$ which is the probability that the diagonal line has the length L. ENTR reflects the complexity of RP in respect of the diagonal line. The value of ENTR is small if the process is noisy.
  4. The average length of the diagonal lines (*L*):
    $$L=\frac{\sum_{l=l_{min}}^{N}l\;p\left(l\right)}{\sum_{l=l_{min}}^{N}p\left(l\right)}$$
- Permutation Entropy (PE) [54]: PE is a non-parametric time series method which is used to quantify the complexity level of a time series data. Assume a given time series data is denoted by $X=\left\{x_{i}\right\},\;i=1,\ldots N$. Common approaches in time series data analysis do not take into consideration the effect of the temporal order of the values in the successive $x_{i}$. To address this issue, the time series can be encoded into sequences of symbols, each one reflecting the rank order of successive xi in sequences of length n. The PE method captures the probability distributions of patterns of symbol sequences, termed permutations. Each activity has different level of complexity. Therefore, measuring the complexity level can be very informative. PE quantifies the complexity by measuring the entropy of sequences:
  $$\mathrm{PE}=-\overset{N}{\underset{j=1}{\sum }}p_{j}^{\prime }\;\ln\left(p_{j}^{\prime }\right)$$
  where $p_{j}^{\prime }$ is the frequency of the possible sequences.
- Lyapunov Exponent (LE) [55]: LE quantifies the rate of divergence of nearby trajectories in the phase space of a dynamic system, which has been proven to be the most useful method in diagnosing the chaos in a dynamical system. A positive value of LE (λ) means that the orbits are chaotic, a zero value indicates that orbits retain in the same position, while a negative value demonstrates that orbits retain their relative positions. Lyapunov exponents quantify the exponential divergence of initially close state-space trajectories and estimate the amount of chaos in a system. In this study a small data quantity method is employed to calculate the largest Lyapunov exponent (LLE) [55]. The algorithm is described as follows:
  $$\lambda =\frac{1}{t_{M}-t_{0}}\overset{M}{\underset{k=1}{\sum }}log_{2}\frac{L^{\prime }\left(t_{k}\right)}{L^{\prime }\left(t_{k-1}\right)}$$
  where $L^{\prime }\left(t_{k}\right)$ denotes the distance between two points in the phase space at time $t_{k}$.
  In this study, a small data quantity method is employed to calculate the largest Lyapunov exponent (LLE) [56]. The algorithm is described as follows: Suppose that the time series acceleration data is $X=\left\{x_{1},x_{2},\ldots .,x_{N}\right\}$, the face space can be represented using the following equation:
  $$Y_{1}=\left\{x_{1},x_{1+\tau },x_{1+2\tau },\ldots \ldots ,x_{1+\left(m-1\right)\tau }\right\}\;Y_{2}=\left\{x_{2},x_{2+\tau },x_{2+2\tau },\ldots \ldots ,x_{2+\left(m-1\right)\tau }\right\}\;:\;:\;Y_{j}=\left\{x_{j},x_{j+\tau },x_{j+2\tau },\ldots \ldots ,x_{j+\left(m-1\right)\tau }\right\}$$
  where τ is the lag, and m is the embedding dimension. After the reconstruction of the dynamics, the algorithm searches for the point that minimizes the distance to the particular reference point. Then, $L^{\prime }\left(t_{k}\right)$, and $L^{\prime }\left(t_{k-1}\right)$ are calculated and used to find the value of λ. 
  The Lyapunov Exponent is calculated with the assumption that the acceleration signal of each feature has a different level of chaos.
- Total Harmonic Distortion (THD) [57]: THD quantifies the distortion of a waveform relative to a pure fundamental frequency. The THD present in a signal is the ratio between square root of the sum of the powers of all harmonic components’ values to the power of the fundamental frequency.
  $$\mathrm{THD}=\frac{\sqrt{\sum_{n=2}^{\infty }V_{n}^{2}}}{V_{fund\;}}$$
  where $V_{n}^{2}$ is the Root mean square (RMS) amplitude of the nth harmonic, and $V_{fund\;}$ is the RMS of the fundamental frequency. In this study only the five first harmonics are included.

Table 2 shows the full list of the extracted features. These features were extracted from each acceleration component of each signal segment. They were also extracted from the signal magnitude ($MG=\sqrt{x^{2}+y^{2}+z^{2}}$). Each window is represented by 193 features in the feature matrix, all of which are calculated for each segment of each axis.

**Figure 2:**
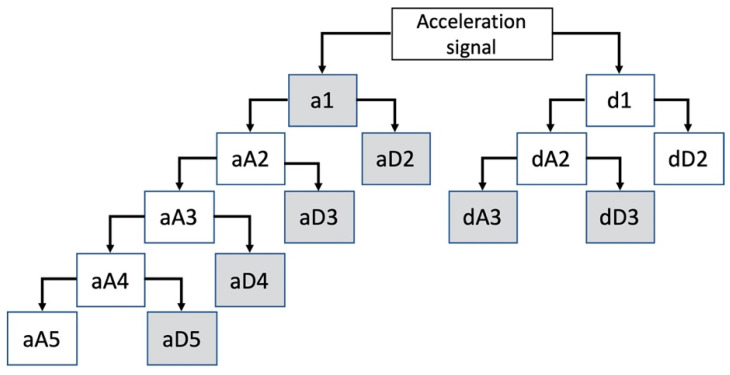
Wavelet packet decomposition tree applied on the signals. Shaded nodes show the targeted coefficients for feature extraction.

In addition to the features discussed earlier, common features include mean, standard deviation, root mean square value, autocorrelation of each signal, Fast Fourier Transform (FFT), Wavelet Packet Decomposition (WPD), power peak features, and autocorrelation. All the features were calculated after the filtration except the mean and standard deviation, which were calculated using the raw data. Many FFT coefficients capture the high frequency part of the signal, these coefficients are likely to have low amplitude which are unlikely to carry significant information about the activity. Most of the information is contained below the frequency of 17 Hz. Activities like walking are low frequency activities. Therefore, WPD shown in Figure 2 is applied to the acceleration signal with decomposition at five levels to capture the lower end of the signals that contain significant information about the activities. A set of features extracted from the coefficients that are shown in Figure 2 are used extract 36 features which include: the sum of absolute value of coefficients from level 1 to 5, energy, and entropy of each acceleration signal along each axis (x,y,z) and MG.

To simplify the presentation of the results, the features are divided into five groups, denoted by giving each group a different colour in Table 2. These groups include simple time domain features, frequency domain features, time-frequency domain features (WPD), repeatability and regularity-based features, and autorepression-based features.

All the features were normalized in order to minimize the effect of any abnormal sensor readings. This can reduce the weight of abnormally high values among the data. Normalization was performed using the following equation:

(1) $$f_{i}=\frac{f_{i}-mean\left(f\right)}{standard\;deviation\left(f\right)}$$

Continuous features in the data were discretised using the Equal Width Discretisation (EWD) method [50] to avoid any bias during the feature selection task. 

#### 2.2.3. Feature Selection 

Feature selection is a dimensionality reduction technique. The current study employs a subset of this technique for two reasons. First, it helps to reduce dimensionality and improves the performance of the learning algorithm by removing the redundant and irrelevant features that may confuse the learning algorithm. Second, it measures the discriminative power of each feature and selects the most informative that produces the highest classification accuracy. The JMIM method was also employed in this research to select the significant feature subset for the discrimination task. This method was chosen, as it was proved that the method outperformed other state-of-the-art methods in the literature, and because it is computationally inexpensive. 

JMIM is based on the use of mutual information and the ‘maximum of the minimum’ criterion. The method starts with the most significant feature, then it begins to select features one by one and add them into the selected subset. The feature is selected when it maximises the goal function:

(2) $$f_{JMIM}=\arg\;max_{f_{i}\in F-S}(min_{f_{s}\in S}\left(I\left(f_{i},f_{s};C\right)\right))$$

where *F* is the original feature set, S is the selected subset feature, $f_{I}$ is the candidate feature and $f_{s}\;$is the selected feature.

The method selects the features that share the highest information with the class label in the context of the features that have already been selected. The method employs the following forward greedy search algorithm:

1. (Initialisation) Set F← “initial set of n features”; S← “empty set.”
2. (Computation of the MI with the output class) For $\forall f_{i}\;\epsilon \;F$ compute $I\left(C;\;f_{i}\right).$
3. (Choice of the first feature) Find a feature $f_{i}\;$that maximises$\;I\left(C;\;f_{i}\right)$; set $F\;\leftarrow \;F\backslash \left\{f_{i}\right\};$ set $S\;\leftarrow \;\left\{f_{i}\right\}$.
4. (Greedy selection) Repeat until|S|=k: (Selection of the next feature) Choose the feature$\;f_{i}=\arg\;max_{f_{i}\in F-S}(min_{f_{s}\in S}\left(I\left(f_{i},f_{s};C\right)\right))$; set $F\;\leftarrow \;F\;\backslash \;\left\{f_{i}\right\};$ set $S\;\leftarrow S\cup^{\;}\left\{f_{i}\right\}$.
5. (Output) Output the set S with the selected features.

The greedy algorithms may not find the optimal solution for some of the heuristic problems when each node in the problem has a fixed value. However, that is not the case for mutual information-based feature selections methods. Although the amount of information between each feature and the class label is theoretically fixed, when the feature is within a subset then this amount of information is no longer fixed, as there is factor called information interaction between the features that can reduce or increase this amount of information. Moreover, the feature may share redundant information that is covered by other features. In addition, the used goal function does not include any summation, it employs maximum of the minimum criterion. Therefore, the search for the features is not as simple as the common heuristic problems. 

The traditional understanding of local minima problem does not apply here because of the information redundancy and information interaction between features which makes everything dynamic with the searched features. 

#### 2.2.4. Classification 

The key point of this work is to investigate the significance of the accelerometer signal features. Classification accuracy is used to evaluate the significance of the selected subset of features. For this reason, two classification algorithms have been used; K-nearest neighbour classifier (KNN) (k = 3), and the Support Vector Machine (SVM) classifier with linear kernel; these classifiers have been chosen because they make few (if any) assumptions about the data. Therefore, the performance will be mainly due to the feature selection not the classifier’s ability to handle the data. They were also chosen because they have been used in similar studies [58].

We used the Matlab Statistics Toolbox for SVM and KNN classifiers. Five-fold cross validation was used for the feature’s significance evaluation. Each fold of the dataset (20%) was used for the validation once, while the rest of the data (80%) were used for the classifiers. This process was repeated five times, which means that the whole dataset was used for the validation. 

The selected subset of features was evaluated after adding each new feature, starting with only the most significant feature in the selected subset. This continued until all 193 features were used together for the training and testing. The classification accuracy produced reflected the discriminative power of the whole subset after adding the new selected feature and was not based on the newly selected feature only. 

## 3. Results

This section describes the results of applying the proposed framework to define the significant feature selection for each dataset. 

### 3.1. Significant Features 

The JMIM method has been used to define the most significant features when classifying the activity within the datasets. This method is classifier independent: it measures the shared information between the class label and the subset of features. Therefore, the features are ranked based on the amount of information that they add about the class, as the selected features add more information when classification accuracy improves. 

The results of the WISDM dataset show that the Root Mean Square (RMS) features shared the highest information with class. Mean, FFT, autocorrelation and wavelet decomposition were among the most significant as well. These five features make up 29 out of the most significant 30 features. 

The RMS feature is shown as the most significant feature in the results of SBRHA dataset. The results also showed that Mean, FFT, autocorrelation and wavelet decomposition are also among the most significant features. The 30 most significant features comprise 28 features from these four feature groups. 

The results of both datasets show that uncommon features, such as RQA, permutation entropy, Lyapunov exponent and total harmonic disorder were not among the most significant features for these two datasets. 

The results of the LG watch and Samsung watch datasets (HARDS) are slightly different. Features that measure the recurrence, regularity and degree of chaos were shown to be among the ten most significant features. These two datasets use relatively high sampling frequencies, which provide more values and more information about the details of the movement. Therefore, features related to repeatability, regularity and level of chaos appeared in the ten most significant features especially for the LG watch data which has a higher sampling frequency, 18 of the 30 most significant features belong to RQA, Permutation entropy, and Lyapunov exponent. Wavelet features also appeared within this list five times. While for the Samsung watch 11 of the 30 most significant features were wavelet features; repeatability related features appeared 10 times in this list. The most significant features were the standard deviation features for both datasets. Many of the twenty most significant features belong to wavelet decomposition features. 

Due to the unique characteristics of PAMAP2 dataset in terms of the number of activities and the level of movement complexity of these activities, the order of the ranked features is different. Features related to Recurrence rate in the signal, WPD, permutation entropy, and autorepression make up most of the features that produce the highest classification accuracy. On the other hand, the results were different when the ankle dataset was down sampled to 20 Hz. The results showed the simple time domain features such mean, and frequency domain features such FFT, WPD, and autorepression features are among the most significant features. Features related to recurrence rate in the signal and permutation entropy appeared less significant when the sampling frequency was reduced. Therefore, features related to Recurrence rate in the signal, wavelet decompensation, permutation entropy can be important for discriminating the activities if the sampling frequency is high and more samples are available for each window. The results also showed that there is no major effect of the sensor placement location on the order of the selected features.

### 3.2. Classification 

Two classifiers were used to evaluate the significance of the ranked selected subset of features for the classification accuracy. As mentioned earlier, each classifier was trained and tested after adding every single feature to the subset. Five-folds cross validation was used for every training and testing iteration. Classification accuracy and F-measure are used to measure the performance of the selected subset of features. In the binary classification, classes are named positive and negative. The confusion matrix describes the ways in which the classifier is confused when making predictions. The confusion matrix has four values representing the relation between real and predicted classes: namely: 

TP (true positives): the number of positive elements classified correctly.

FP (false positives): the number of negative elements misclassified as positive.

FN (false negatives): the number of positive elements misclassified as negative.

TN (true negatives): the number of negative elements misclassified as negative.

$$Classification\;accuracy=\frac{TP+TN}{TP+TN+FP+FN}$$

$$Precision=\frac{TP}{TP+FP}$$

$$Recall=\frac{TP}{TP+FN}$$

$$F-measure=2\times \frac{Precision\times Recall}{Precision+Recall}$$

Extending these classification metrics to multi-class problems can be achieved by considering one of the classes as a positive class and the remaining ones as negative class [59]:

$$Classification\;accuracy=\frac{1}{M}\overset{M}{\underset{i=1}{\sum }}\frac{TP_{i}+TN_{i}}{TP_{i}+TN_{i}+FP+FN_{i}{}_{i}}$$

$$Precision=\frac{1}{M}\overset{M}{\underset{i=1}{\sum }}\frac{TP_{i}}{TP_{i}+FP_{i}}$$

$$Recall=\frac{1}{M}\overset{M}{\underset{i=1}{\sum }}\frac{TP_{i}}{TP_{i}+FN_{i}}$$

where M is the number of distinct classes in the datasets.

**Figure 3:**
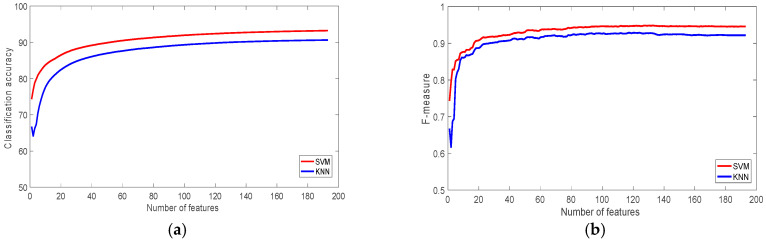
The performance of the classifiers with the WISDM Dataset: (**a**) Classification accuracy; (**b**) F-measure.

Figure 3 shows the classification accuracy and f-measures figures of the WISDM dataset using SVM and KNN classifiers. It is clear that SVM outperformed KNN and achieved 93% accuracy, while KNN achieved 90.65%. Table 3 shows the classification accuracy using 1, 10, 30, 60, 120 and 193 ranked features. The table shows that the most significant feature on its own is able to produce more than 74% accuracy, which is about 80% of the classification accuracy achieved using all 193 features. With just 30 features, the classification accuracy of the SVM classifier was more than 94% of the accuracy of the total features. The achieved accuracy outperforms the accuracy of the original study, which used the same dataset [60], which was 91.7%. 

The results of the SBRHA dataset showed that with only the most significant feature, the SVM classifier was able to classify the activities with the different accuracy; jogging (93.8%), walking (67.89%), sitting (95%), standing (89.46%), while the classifier missed classifying all the walking upstairs and downstairs activities. The results of using the subset of 30 features showed that walking upstairs and downstairs are also the most misclassified, as walking and jogging have an accuracy of (57.55%), while standing is with the highest accuracy (99%), then jogging (97.02%), walking (92.92%) and sitting (95%).

**Figure 4:**
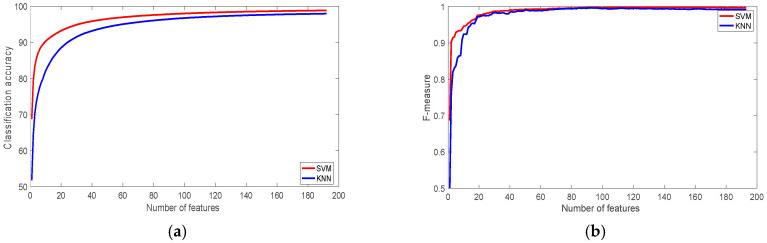
The performance of the classifiers with the SBRHA Dataset: (**a**) Classification accuracy; (**b**) F-measure.

Although the dataset is not balanced, Figure 4 shows that f-measure is relatively high for both classifiers, the value of f-measure when the most significant 30 features have used to train the classifiers was 0.91, and 0.90 for SVM and KNN respectively.

**Figure 5:**
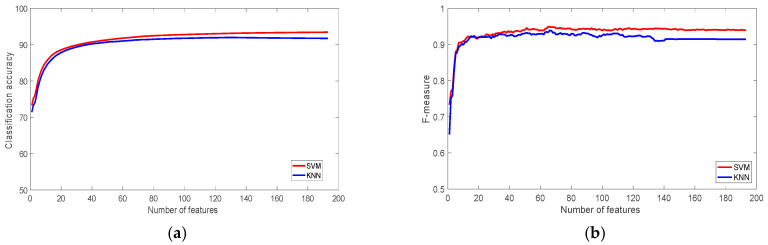
The performance of the classifiers with the HARDS Dataset (LG): (**a**) Classification accuracy; (**b**) F-measure.

As noted earlier, only the data collected by the smart watches from HARDS dataset was used in this study, the rest of HARDS dataset was not included. Figure 5 shows the results of the classification accuracy of the LG watch with the most significant subset of 30 features was 89.87%, and 89.01% for SVM and KNN classifiers respectively. The f-measure with the same number of significant features was 0.92 for both classifiers. Laying down is the activity that was classified least correctly with a recognition rate of 80.68%; it was misclassified as standing and sitting. This accuracy stayed almost steady after the feature subset reached 120 features, which means the rest of the features were redundant, the accuracy for SVM, and KNN were 93.05%, and 91.84% respectively.

In terms of classification rate of each activity when the most 120 significant features are used, biking scored the highest classification (98.74%) followed by sitting (98.73%), standing (92.91%), walking (88.18%), and walking up and down (84.06%). This order stays almost the same when the most 30 significant features were used, the only difference is swapping the order between walking and walking up the stairs.

**Figure 6:**
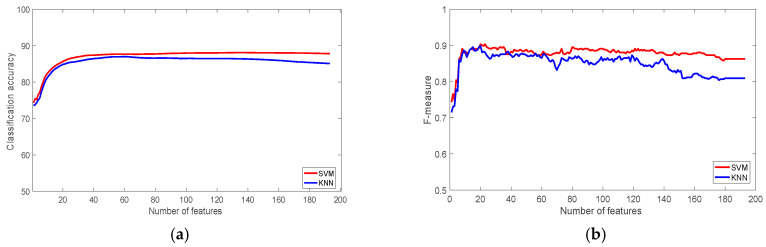
The performance of the classifiers with the HARDS Dataset (Samsung Watch): (**a**) Classification accuracy; (**b**) F-measure.

The results of the Samsung watch dataset are shown in Figure 6. They show that the classification accuracy and f-measures of were lower than the LG watch. The highest classification accuracy was 88% and 86% for SVM, and KNN, respectively, which was achieved using the 120 most significant features. At the same time the f-measure using this subset of features was 0.88, and 0.85 for the SVM, and KNN classifiers. The figures of this dataset agree with the previous dataset about the redundancy of the 70 least significant features. The walking up the stairs activity had the lowest recognition rate of 58.70%, it was again misclassified also as standing and walking.

**Figure 7:**
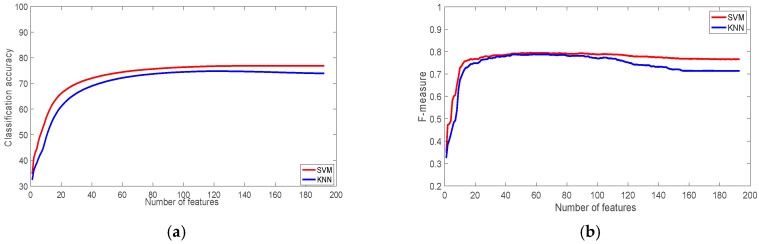
The performance of the classifiers with the PAMAP2 Dataset (Ankle): (**a**) Classification accuracy; (**b**) F-measure.

**Figure 8:**
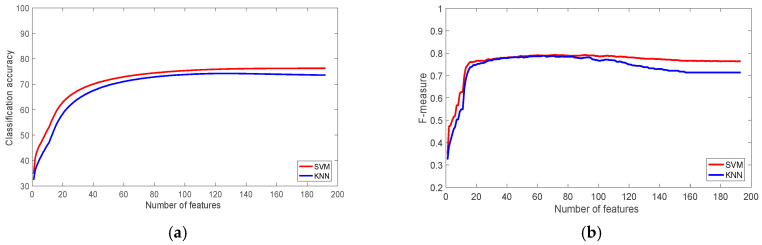
The performance of the classifiers with the PAMAP2 Dataset (Chest): (**a**) Classification accuracy; (**b**) F-measure.

**Figure 9:**
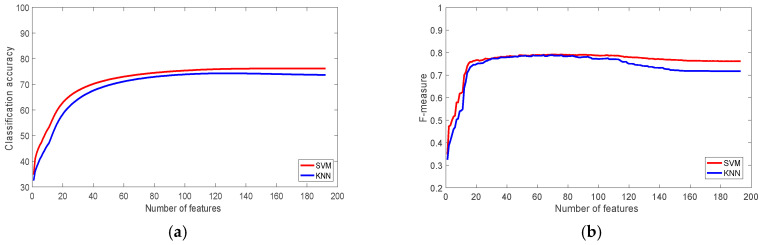
The performance of the classifiers with the PAMAP2 Dataset (Hand): (**a**) Classification accuracy; (**b**) F-measure.

The results for the PAMAP2 datasets for different sensor placement locations are shown in Figure 7, Figure 8 and Figure 9. The results shows that there is no significant influence of the sensor placement on the classification accuracy. However, there is a slight advantage when sensors are placed on the ankle. Due to the high number of activities in the PAMAP2 datasets, some of which consist of complex movements, the classification accuracy for PAMAP2 datasets is slightly lower than the other datasets used in this study. For the ankle dataset, walking up and down the stairs were the most misclassified activities with a recognition rate of 40.71%, and 57.64%. They were misclassified as walking, running, and ironing activities.

To evaluate the discriminative power of the uncommon features, which are the features related to RQA, PE, LE, and THE, the classification algorithms were trained without using the uncommon features. The drop of the classification accuracy is used as indication of the discriminative power of these features. Table 4 illustrate the results of this experiment, 

Adding the uncommon features to the dataset enhanced the classification accuracy by 2.3–8.6%. The results showed that the enhancement is larger when the sampling frequency is higher, which means the uncommon features are able to extract more patterns when the sampling frequency is high. 

## 4. Discussion 

The significance of accelerometer features was successfully identified using the state-of-the-art JMIM feature selection method. The evaluation of the selected subset was carried out using SVM and KNN classifiers. Four publicly available datasets were used in this study because they have been previously used in activity recognition research and also to study the effect of sampling frequency as well as on body placement location on the feature significance for classification accuracy. The sampling frequency of the datasets is between 20 and 200 Hz. Wearables are placed at the waist, wrist, chest, and ankle. The number of activities also vary, with most of the datasets including only simple activities, and one of them including complex activities.

This study focused on the type of the features rather than the acceleration axis to which the signal belongs. The features are divided into five groups: (simple time domain), (frequency domain which includes FFT, and spectral features), (repeatability and regularity which includes RQA, permutation entropy, and Lyapunov exponent), (time-frequency which includes WPD), and finally (autorepression features).

The results showed that the Root Mean Square (RMS) features produced 74% accuracy on their own for the WISDM and SBRHA datasets. FFT, Root Mean Square (RMS), mean, autocorrelation and wavelet were among the most significant features for human activity recognition for these two datasets. This result agreed with the results in previous literature [14].

On the other hand, when the sampling frequency is raised, features related to signal repeatability, regularity and level of chaos (RQA, Permutation Entropy, Lyapunov exponent), and WPD were shown among the most significant. The sampling rate was 100 and 200 Hz, which meant that each contained more data points. This high number of data points provided more detailed information about the movement. That is why these features appeared among the most significant in the subset. Wavelet decomposition features were also among the most significant features for this dataset. This has been shown in the results from the PAMAP2 and HARDS datasets. 

Even though many participants were recruited to perform the activities during the data collection, which meant that the activities were performed at different speeds and styles, the achieved classification accuracy was very high, especially for SBRHA (more than 98%). The results also showed that SVM worked better than KNN.

In conclusion, simple time domain features can be very predictive for recognising simple activities, as the sampling frequency becomes high WPD, and repeatability and regularity features can be very predictive, especially when the recognised activities include complex activities. WPD features can have significant discriminative power for recognising both simple and complex activities. 

It would be interesting in the future to study the influence of window size and overlapping on the selected feature subset, as well as the effect of applying invariant orientation techniques on the feature significance. Further improvements are needed for the selection method to be able to produce scores for significance degrees. 

Future research could also be conducted using new collected data with more activities, and more participants could be included. In particular, datasets should report full demographic statistics for participants including at least age, gender, ethnicity, and body mass index. The public datasets have poor descriptions of their participant demographics with only one reporting the number of females (1/9), thus it is likely that the datasets are heavily biased towards young white men. Future public datasets should reflect the variation in real world, including additional participants from diverse age groups and with different ranges of movement capabilities in order to study the feature significance for data collected from a variety of populations, and different patients with movement impairments. A future study can be expanded to include all IMU sensors, which will require studying the gyroscope drift problem, and comparing various compensation approaches. The methodology of this study can also be employed to study controlling powered exoskeletons through developing machine learning-based algorithms to recognise the movement.

## Figures and Tables

**Table 1:** Datasets Used in the Experiment.

Dataset	Number of Instances	Activities	Sampling Frequency (Hz)	Placement Location	Sensor
WISDM	1,098,207.00	Walking (38.6%) Jogging (31.2%) Upstairs (11.2%) Downstairs (9.1%) Sitting (5.5%) Standing (4.4%)	20	Waist	Smart phone
HARDS	1,048,576.00	Walking (18.10%) Biking (20.33%) Upstairs (13.03%) Downstairs (13.70%) Sitting (16.03%) Standing (19.24%)	200, and 100	Wrist	Smart-phone and Smart-watch
SBRHA	10,929.00	Walking (16.72%) Upstairs (14.54%) Downstairs (14.12%) Sitting (18.51%) Standing (17.24%) Laying (18.87%)	50	Waist	Smart phone
PAMAP2	3,850,505.00	Lying (6.7%) Sitting (13.06%) Walking (8.2%) Running (3.3%) Cycling (5.73%) Nordic walking (6.48%) Upstairs (4.08%) Downstairs (3.65) vacuum cleaning (6.13%) Ironing (8.31%) Rope jumping (1.92%) Other (transient activities) (32.44%)	100	Wrist, Chest and Ankle	IMUs

**Table 2:** List of Extracted Features.

Feature Number	Feature Description
pi	Mean of acceleration components (x,y,z) and MG
5–8	The standard deviation of each axis (x,y,z) and MG
9–12	Root Mean Square (RMS) value for each component (x,y,z) and MG
13–24	The autocorrelation of each signal, 3 for each component (x,y,z) and MG
25–72	Spectral peaks features, height and position of first 6 peaks of each component (x,y,z), and MG
73–84	Total power in 3 adjacent and pre-defined frequency bands (x,y,z) and MG
85–100	The magnitude of first 3 Fast Fourier Transform (FFT) components and the FFT entropy for each axis (x,y,z) and MG
101–136	The sum of absolute value of wavelet packet decomposition coefficients from level 1 to 5, Energy, and entropy (x,y,z) and MG
137–139	The first principal component of PCA for each component (x,y,z) and MG
140–143	RQA features for X axis.
144	Lypaunov exponent for component x
145	Permutation entropy for component x
146–149	RQA features for Y axis.
150	Lypaunov exponent for component y
151	Permutation entropy for component y
152–155	RQA features for Z axis.
156	Lypaunov exponent for component z
157	Permutation entropy for component z
158–161	RQA features for MG signal
162	Lypaunov exponent for component MG signal
163	Permutation entropy for component MG signal
164–167	Total Harmonic Distortion of each component (x,y,z) and MG
168	$\mathrm{Maximum}\;\mathrm{Difference}\;Dxyz=\sqrt{(dx^{2}+dy^{2}+dz^{2}})$
169	Total acceleration signal magnitude area
170–193	Autoregressive coefficient of order 5 model for each component (x,y,z) and MG

**Table 3:** Classification Accuracy with 1, 10, 30, 60, 120 and 193 Ranked Features.

Number of Ranked Features	SVM							KNN						
	WISDM	SBRHA	HARDS		PAMAP2			WISDM	SBRHA	HARDS		PAMAP2		
			LG Watch	Samung Watch	Ankle	Chest	Hand			LG Watch	Samsung Watch	Ankle	Chest	Hand
1	74.33	68.66	73.31	74.30	34.77	34.80	34.83	66.74	51.70	71.45	73.53	32.44	32.45	32.47
10	83.78	90.29	85.14	82.41	56.38	52.06	52.29	77.68	82.06	83.84	81.27	48.81	46.12	46.20
30	88.20	94.84	89.89	86.94	69.90	67.46	67.54	84.76	91.49	89.35	85.72	66.16	64.18	64.17
60	90.49	96.93	91.79	87.65	74.42	72.89	72.97	87.63	95.00	91.00	86.98	72.16	71.03	71.09
120	92.40	98.26	93.03	88.01	76.70	75.85	75.89	89.82	97.15	91.93	86.45	74.77	74.18	74.28
193	93.24	98.81	93.45	87.80	76.85	76.23	76.20	90.65	97.94	91.74	85.09	73.87	73.54	73.64

**Table 4:** Classification Accuracy without, and with uncommon features using SVM classifier.

HARDS		WISDM		HARDS				PAMAP2					
				LG		Samsung		Ankle		Chest		Hand	
Without	With	Without	With	Without	With	Without	With	Without	With	Without	With	Without	With
90.2%	98.8%	88.9%	93.2%	90.8%	93.4%	85.4%	87.8%	70.51%	76.8%	72.9%	76.2%	70.5%	76.2%

## Data Availability

The Publicly available dataset that were used in this study can be found at these links: 1. **WISDM:** https://archive.ics.uci.edu/ml/datasets/WISDM+Smartphone+and+Smartwatch+Activity+and+Biometrics+Dataset+; 2. **HARDS**: https://archive.ics.uci.edu/ml/datasets/heterogeneity+activity+recognition; 3. **SBRHA:** https://archive.ics.uci.edu/ml/datasets/smartphone-based+recognition+of+human+activities+and+postural+transitions; 4. **PAMAP2:** https://archive.ics.uci.edu/ml/datasets/pamap2+physical+activity+monitoring.

## Abbreviations

AAL	Ambient Assistive Living
ADL	Activities of Daily Living
CFS	Feature Selection
DET	Determinism
ENTR	Entropy
FCBF	Fast Correlation Based Filter
FCBF	Fast Correlation Based Filter
FFT	Fast Fourier Transform
GRRF	Guided Regularized Random Forest
HA	Human Activity
HARDS	Heterogeneity Activity Recognition Dataset
IMU	Inertial Measurement Unit
JMIM	Joint Mutual Information Maximisation
KNN	K-Nearest Neighbor
LDA	Discriminant Analysis
LE	Lyapunov Exponent
mRMR	minimum Redundancy Maximum Relevance
PA	Physical Activity
PAMAP2	Physical Activity Monitoring for Aging People Dataset
PCA	Principal Component Analysis
PE	Permutation Entropy
RMS	Root Mean Square
RQA	Recurrence Quantification Analysis
RR	Recurrence Rate
SBRHA	Smartphone-Based Recognition of Human Activities and Postural Transitions Dataset
SFFS	Sequential Forward Floating Search
SVD	Singular Value Decomposition
SVM	Support Vector Machine
THD	Total Harmonic Distortion
WISDM	WIreless Sensor Data Mining Dataset
WPD	Wavelet Packet Decomposition

## References

[1] Zdravevski E., Stojkoska B.R., Standl M., Schulz H. (2017). Automatic machine-learning based identification of jogging periods from accelerometer measurements of adolescents under field conditions. PLoS ONE.

[2] World Health Organization (2010). Global Recommendations on Physical Activity for Health.

[3] United Nations, Department of Economic and Social Affairs, Population Division (2017). World Population Prospects 2017—Data Booklet (ST/ESA/SER.A/401).

[4] King R.C., Villeneuve E., White R.J., Sherratt R.S., Holderbaum W., Harwin W.S. (2017). Application of data fusion techniques and technologies for wearable health monitoring. Med. Eng. Phys..

[5] Attal F., Mohammed S., Dedabrishvili M., Chamroukhi F., Oukhellou L., Amirat Y. (2015). Physical human activity recognition using wearable sensors. Sensors.

[6] Washburn R.A., Smith K.W., Jette A.M., Janney C.A. (1993). The Physical Activity Scale for the Elderly (PASE): Development and evaluation. J. Clin. Epidemiol..

[7] Mubashir M., Shao L., Seed L. (2013). A survey on fall detection: Principles and approaches. Neurocomputing.

[8] Godfrey R., Conway D., Meagher G. (2008). OLaighin Direct measurement of human movement by accelerometry. Med. Eng. Phy..

[9] Islam R., Holland S., Price B., Georgiou T., Mulholland P. Wearables for Long Term Gait Rehabilitation of Neurological Conditions. Proceedings of the Short Workshop on Next Steps Towards Long Term Self Tracking, CHI 2018: CHI Conference on Human Factors in Computing Systems.

[10] Bennasar M., Hicks Y.A., Clinch S.P., Jones P., Holt C., Rosser A., Busse M. (2018). Automated Assessment of Movement Impairment in Huntington’s Disease. IEEE Trans. Neural Syst. Rehabil. Eng..

[11] Mohammad Y., Matsumoto K., Hoashi K. (2019). Selecting orientation-insensitive features for activity recognition from accelerometers. IEICE Trans. Inf. Syst..

[12] Mohsen S., Elkaseer A., Scholz S.G. (2021). Industry 4.0-oriented deep learning models for human activity recognition. IEEE Access.

[13] Bordel B., Alcarria R., Robles T. (2022). Recognizing human activities in Industry 4.0 scenarios through an analysis-modeling-recognition algorithm and context labels. Integr. Comput.-Aided Eng..

[14] Twomey N., Diethe T., Fafoutis X., Elsts A., McConville R., Flach P., Craddock I. (2018). A comprehensive study of activity recognition using accelerometers. Informatics.

[15] Bao L., Intille S.S. Activity recognition from user-annotated acceleration data. Proceedings of the International Conference on Pervasive Computing.

[16] Maurer U., Sudderth E.B., Jordan M.I., Willsky A.S. Activity recognition and monitoring using multiple sensors on different body positions. Proceedings of the International Workshop on Wearable and Implantable Body Sensor Networks (BSN).

[17] Sztyler T., Stuckenschmidt H. On-body localization of wearable devices: An investigation of position-aware activity recognition. Proceedings of the 2016 IEEE International Conference on Pervasive Computing and Communications (PerCom).

[18] Woznowski P., Kaleshi D., Oikonomou G., Craddock I. (2016). Classification and suitability of sensing technologies for activity recognition. Comput. Commun..

[19] Capela N.A., Lemaire E.D., Baddour N. (2015). Feature selection for wearable smartphone-based human activity recognition with able bodied, elderly, and stroke patients. PLoS ONE.

[20] Lara O.D., Labrador M.A. (2013). A survey on human activity recognition using wearable sensors. IEEE Commun. Surv. Tutor..

[21] Bersch S.D., Azzi D., Khusainov R., Achumba I.E., Ries J. (2014). Sensor data acquisition and processing parameters for human activity classification. Sensors.

[22] Bennasar M., Hicks Y., Setchi R. (2015). Feature selection using joint mutual information maximisation. Expert Syst. Appl..

[23] Turk M., Pentland A. (1991). Eigen faces for recognition. J. Cogn. Neurosci..

[24] Yu H., Yang J. (2001). A direct LDA algorithm for high-dimensional data with application to face recognition. Pattern Recognit..

[25] Krause A., Siewiorek D.P., Smailagic A., Farringdon J. Unsupervised, dynamic identification of physiological and activity context in wearable computing. Proceedings of the 2012 16th International Symposium on Wearable Computers: IEEE Computer Society.

[26] Bennasar M., Setchi R., Hicks Y. (2013). Feature interaction maximisation. Pattern Recognit. Lett..

[27] Micucci D., Mobilio M., Napoletano P. (2017). UniMiB SHAR: A Dataset for Human Activity Recognition Using Acceleration Data from Smartphones. Appl. Sci..

[28] Vail D.L., Veloso M.M., Lafferty J.D. Conditional random fields for activity recognition. Proceedings of the 6th International Joint Conference on Autonomous Agents and Multiagent Systems.

[29] Chathuramali K.M., Rodrigo R. Faster human activity recognition with SVM. Proceedings of International Conference on Advances in ICT for Emerging Regions (ICTer2012).

[30] Cilla R., Patricio M.A., García J., Berlanga A., Molina J.M. (2009). Recognizing human activities from sensors using hidden markov models constructed by feature selection techniques. Algorithms.

[31] Zeng Z., Ji Q. Knowledge based activity recognition with dynamic bayesian network. Proceedings of the European Conference on Computer Vision.

[32] Anguita D., Ghio A., Oneto L., Parra Perez X., Reyes Ortiz J.L. A public domain dataset for human activity recognition using smartphones. Proceedings of the 21th International European Symposium on Artificial Neural Networks, Computational Intelligence and Machine Learning.

[33] Khan A., Hammerla N., Mellor S., Plötz T. (2016). Optimising sampling rates for accelerometer-based human activity recognition. Pattern Recognit. Lett..

[34] Preece S.J., Goulermas J.Y., Kenney L.P., Howard D. (2008). A comparison of feature extraction methods for the classification of dynamic activities from accelerometer data. IEEE Trans. Biomed. Eng..

[35] Gupta P., Dallas T. (2014). Feature selection and activity recognition system using a single triaxial accelerometer. IEEE Trans. Biomed. Eng..

[36] Atallah L., Lo B., King R., Yang G.Z. Sensor placement for activity detection using wearable accelerometers. Proceedings of the 2010 International Conference on Body Sensor Networks.

[37] Chernbumroong S., Atkins A.S., Yu H. Activity classification using a single wrist-worn accelerometer. Proceedings of the 5th International Conference on Software, Knowledge Information, Industrial Management and Applications (SKIMA).

[38] Suto J., Oniga S., Sitar P.P. (2017). Feature analysis to human activity recognition. Int. J. Comput. Commun. Control..

[39] Yang A.Y., Kuryloski P., Bajcsy R. WARD: A Wearable Action Recognition Database. Proceedings of the CHI 2009: CHI Conference on Human Factors in Computing Systems.

[40] Rosati S., Balestra G., Knaflitz M. (2018). Comparison of different sets of features for human activity recognition by wearable sensors. Sensors.

[41] Orlov A.A., Makarov K.V., Tarantova E.S. Features Selection for Human Activity Recognition in Telerehabilitation. Proceedings of the 2019 International Science and Technology Conference “EastConf”.

[42] Ahmed N., Rafiq J.I., Islam M.R. (2020). Enhanced Human Activity Recognition Based on Smartphone Sensor Data Using Hybrid Feature Selection Model. Sensors.

[43] Thakur D., Biswas S. (2022). An Integration of feature extraction and Guided Regularized Random Forest feature selection for Smartphone based Human Activity Recognition. J. Netw. Comput. Appl..

[44] Dua D., Karra Taniskidou E. (2017). UCI Machine Learning Repository.

[45] Kwapisz J.R., Weiss G.M., Moore S.A. (2011). Activity recognition using cell phone accelerometers. ACM SigKDD Explor. Newsl..

[46] Reiss A., Stricker D. Creating and benchmarking a new dataset for physical activity monitoring. Proceedings of the 5th International Conference on PErvasive Technologies Related to Assistive Environments.

[47] Anguita D., Ghio A., Oneto L., Parra X., Reyes-Ortiz J.L. Human activity recognition on smartphones using a multiclass hardware-friendly support vector machine. Proceedings of the International workshop on ambient assisted living.

[48] Stisen A., Blunck H., Bhattacharya S., Prentow T.S., Kjærgaard M.B., Dey A., Sonne T., Jensen M.M. Smart devices are different: Assessing and mitigating mobile sensing heterogeneities for activity recognition. Proceedings of the 13th ACM Conference on Embedded Networked Sensor Systems.

[49] Bruno B., Mastrogiovanni F., Sgorbissa A., Vernazza T., Zaccaria R. Human motion modelling and recognition: A computational approach. Proceedings of the IEEE International Conference on Automation Science and Engineering (CASE).

[50] Wang G., Li Q., Wang L., Wang W., Wu M., Liu T. (2018). Impact of sliding window length in indoor human motion modes and pose pattern recognition based on smartphone sensors. Sensors.

[51] Głowiński S., Ptak M. (2022). A kinematic model of a humanoid lower limb exoskeleton with pneumatic actuators. Acta Bioeng. Biomech..

[52] Webber Jr C.L., Zbilut J.P. (2005). Recurrence quantification analysis of nonlinear dynamical systems. Tutor. Contemp. Nonlinear Methods Behav. Sci..

[53] Eckmann J.P., Kamphorst S.O., Ruelle D. (1995). Recurrence plots of dynamical systems. World Sci. Ser. Nonlinear Sci. Ser. A.

[54] Riedl M., Müller A., Wessel N. (2013). Practical considerations of permutation entropy. Eur. Phys. J. Spec. Top..

[55] Wolf A., Swift J.B., Swinney H.L., Vastano J.A. (1985). Determining Lyapunov exponents from a time series. Phys. D Nonlinear Phenom..

[56] Rosenstein M.T., Collins J.J., De Luca C.J. (1993). A practical method for calculating largest Lyapunov exponents from small data sets. Phys. D Nonlinear Phenom..

[57] Shmilovitz D. (2005). On the definition of total harmonic distortion and its effect on measurement interpretation. IEEE Trans. Power Deliv..

[58] Dougherty J., Kohavi R., Sahami M. Supervised and unsupervised discretization of continuous features. Proceedings of the Machine Learning.

[59] Gao L., Bourke A.K., Nelson J. A comparison of classifiers for activity recognition using multiple accelerometer-based sensors. Proceedings of the 2012 IEEE 11th International Conference on Cybernetic Intelligent Systems (CIS).

[60] Espíndola R.P., Ebecken N.F.F. (2005). On extending f-measure and g-mean metrics to multi-class problems. WIT Trans. Inf. Commun. Technol..

